# Efficacy of a multifaceted podiatry intervention to improve balance and prevent falls in older people: a randomised trial

**DOI:** 10.1186/1757-1146-4-S1-O47

**Published:** 2011-05-20

**Authors:** Martin J Spink, Mohammad R Fotoohabadi, Elin Wee, Karl B Landorf, Keith D Hill, Stephen R Lord, Hylton B Menz

**Affiliations:** 1 Musculoskeletal Research Centre, Faculty of Health Sciences, La Trobe University, Bundoora, Victoria, 3086, Australia; 2 Department of Podiatry, Faculty of Health Sciences, La Trobe University, Bundoora, Victoria, 3086, Australia; 3 Division of Allied Health, Northern Health, Epping, Victoria, 3076, Australia; 4 Preventive and Public Health Division, National Ageing Research Institute, Parkville, Victoria, 3052, Australia; 5 School of Public Health and Community Medicine, University of New South Wales, Sydney, NSW, 2052, Australia; 6 Neuroscience Research Australia, Randwick, Sydney, NSW, 2031, Australia

## Background

There is growing evidence that foot problems and inappropriate footwear impair balance and increase the risk of falls in older people. These risk factors are potentially amenable by podiatric intervention, although this is yet to be evaluated in a clinical trial. This presentation reports on the first randomised controlled trial of a multifaceted podiatry intervention designed to improve balance and prevent falls in older people.

## Methods

We randomly allocated 305 community-dwelling people aged over 65 years with foot pain and an increased risk of falling to a control or intervention group. Both groups received routine podiatry care. The intervention group also received a multifaceted podiatry intervention consisting of: (i) prefabricated insoles customised to accommodate plantar lesions; (ii) footwear advice and assistance with the purchase of new footwear if current footwear was inappropriate; (iii) a home-based exercise program to strengthen foot and ankle muscles; and (iv) a falls prevention education booklet. Primary outcome measures were the number of fallers/multiple fallers and the falls rate, recorded by a falls diary over a 12 month period. Secondary outcome measures assessed six months after baseline included the Short Form 12 (SF-12), the Manchester Foot Pain and Disability Index (MFDPI), the Falls Efficacy Scale International, foot and ankle strength and range of motion (ROM), and balance and functional tests.

## Results

There was a significant reduction in falls rate in the intervention group compared to the control group (incidence rate ratio=0.64, 95% CI 0.45 to 0.91, p=0.01) but no significant differences between the groups in the proportion of fallers/multiple fallers. There were also significant improvements in secondary outcome measures of the SF-12 physical subscale, the MFPDI function subscale, and several measures of foot and ankle strength and ROM, balance and functional ability in the intervention group compared to the control group.

## Conclusions

In older people with foot pain and an elevated risk of falling, a multifaceted podiatry intervention reduces the rate of falls by 36% and improves several aspects of foot and ankle strength, ROM, balance and functional ability suggesting that podiatry has a valuable role to play in preventing falls in older people.

